# Association between CCN1 gene polymorphism and acute coronary syndrome in Chinese Han and Uygur populations

**DOI:** 10.1186/s41065-021-00180-2

**Published:** 2021-04-27

**Authors:** Yan-Hong Li, Jun-Yi Luo, Bin-Bin Fang, Guo-Li Du, Ting Tian, Fen Liu, Xiao-Mei Li, Yi-Ning Yang

**Affiliations:** 1 Department of Clinical Laboratory, First Affiliated Hospital of Xinjiang Medical University, Urumqi, 830054 China; 2 Department of Cardiology, State Key Laboratory of Pathogenesis, Prevention and Treatment of High Incidence Diseases in Central Asian, First Affiliated Hospital of Xinjiang Medical University, Urumqi, 830054 China; 3 Department of Cardiology, First Affiliated Hospital of Xinjiang Medical University, 137 Liyushan South Road, Urumqi, 830054 Xinjiang China; 4 State Key Laboratory of Pathogenesis, Prevention and Treatment of High Incidence Diseases in Central Asian, First Affiliated Hospital of Xinjiang Medical University, Urumqi, 830054 China; 5 Department of Endocrinology, First Affiliated Hospital of Xinjiang Medical University, Urumqi, 830054 China; 6 People’s Hospital of Xinjiang Uygur Autonomous Region, Urumqi, 830054 China

**Keywords:** Acute coronary syndrome, CCN1, Polymorphism, Genotyping, Haplotype

## Abstract

**Background:**

CCN1 plays a crucial role in the modulation of cardiovascular diseases. However, whether *CCN1* genetic variants are involved in the susceptibility of ACS remains unknown. Hence, the present study investigates the association between *CCN1* polymorphisms and ACS among Han and Uygur populations in Xinjiang, China.

**Results:**

In this case-control study, 1234 Han (547 ACS patients and 687 controls) and 932 Uygur (471 ACS patients and 461 controls) were genotyped using SNPscan^TM^ for three single-nucleotide polymorphisms (SNPs, rs6576776, rs954353, and rs3753794) of the human *CCN1* gene. In the Uygur population, we found that the detected frequencies of the C allele (25.3% vs. 18.3%, *P*<0.001) and CC genotype (6.4% vs. 3.0%, *P*=0.001) of rs6576776 were significantly higher in the ACS patients than in the control participants. Differences in rs6576776 regarding the dominant model (CC+CG vs. GG, 44.2% vs. 55.8%, *P*=0.001) and the recessive model (CC vs. CG+GG, 6.4% vs. 93.6%, *P*=0.016) were observed between the two groups. The frequencies of the GGC and AGC haplotypes in those with ACS were significantly higher than those in the control group (all *P*<0.05) in the Uygur population. After adjusting for hypertension, diabetes, lipids and smoking, all of which indicate that the rs6576776 C allele is associated with higher risk of ACS (odds ratio (OR)=1.798, 95% confidence interval (CI), 1.218-2.656, *P*=0.003). In Han population, neither the distribution of genotypes and alleles of the *CCN1* gene three SNPs nor the distribution of haplotypes constructed with the three SNPs exhibited a significant difference between the ACS patients and control participants.

**Conclusions:**

Our study document that the *CCN1* gene rs6576776 C allele is associated with higher susceptibility of ACS and that the frequencies of GGC and AGC haplotypes are higher among the Uygur ACS patients.

## Background

Coronary artery disease (CAD) is the leading cause of death worldwide. In 2008, more than 17 million people died from CAD and 10% of the global disease burden was attributed to CAD, of which the most common forms are chronic coronary syndrome and acute coronary syndrome (ACS) [1]. Through the destabilization of atherosclerotic plaques, stable CAD can progress into ACS via a group of clinical syndromes of which the pathological basis is atherosclerotic plaque rupture and thrombosis [2]. As the development of ACS is accompanied by high cardiovascular morbidity and mortality, the early identification of patients at risk followed by rapid therapeutic intervention is critical [2].

While inflammatory cells and inflammatory factors can be detected in all stages of atherosclerotic plaque formation, the development of ACS resembles typical acute inflammation with the participation of immune response components. Cysteine-rich 61 (Cyr61/CCN1) belongs to the CCN (CYR61, GIG1 and IGFBP10) family and is a secreted extracellular matrix (ECM) protein which is a novel pro-inflammatory factor [3]. Not only has been reported that CCN1 plays a pivotal role in regulating cholesterol metabolism and the development of atherosclerosis [4], but there is increasing evidence that CCN1 participates in the development and progression of various cardiovascular diseases [5]. CCN1 immunopositivity in most cardiac tissue specimens obtained from young and middle-aged victims of sudden cardiac death has indicated that CCN1 may be associated with ischemic morphology and hypertrophy of myocardial fibers [6]. More specifically, the CCN1 level in CAD patients was significantly higher than that in the controls, and the level of CCN1 was positively correlated with both the Gensini score and the C-reactive protein level [7]. Moreover, the elevated CCN1 levels were significantly associated with the severity of acute heart failure in a cohort of 183 patients [8]. In addition, animal heart failure experiments also revealed that the expression of CCN1 protein was increased [9–11].

Human *CCN1* has been mapped to chromosome 1p22.3 [12]. Among the three SNPs, rs6576776 and rs3753794 locate upstream and downstream transcript variants separately. The polymorphism rs3753794 of the CCN1 gene was shown to be associated with plasma high-density lipoprotein–cholesterol levels in obese individuals [13]. Chang-Chun Niu et al. findings indicated that rs6576776 in the CCN1 gene may be considered potential acute myeloid leukemia risk factors in the Han Chinese population [14]. So far, the function of rs954353 has not been studied at the cellular and animal levels. Recently, studies that have focused on the association between the *CCN1* gene and diseases and have demonstrated that the *CCN1* rs12756618 variant increases the risk of Graves’ ophthalmopathy [15], that the *CCN1* p.R47W variant is related to atrial septal defects [16] and that the *CCN1* rs3753793 variant may contribute to the risk of prostate cancer [17]. Nonetheless, the association of CCN1 polymorphisms and ACS has not been reported. Therefore, we conducted a case-control study to investigate the relation between the *CCN1* gene and the susceptibility of ACS in the Chinese Uygur and Han populations in Xinjiang, China.

## Results

### Demographic data of participants

The separate demographic and clinical characteristics of the study populations of Han and Uygur are presented in Table 1. In the Uygur population, we found that when compared with the control groups (all *P*<0.05), the ACS patients exhibited higher leukocyte (WBC), triglycerides (TG), total cholesterol (TC), low density lipoprotein-cholesterol (LDL-C), creatinine (CREA), uric acid (URIC) and glucose levels, a lower high density lipoprotein-cholesterol (HDL-C) level and DM prevalence. With respect to the Han population, we found that ACS patients had higher levels of WBC, TG, TC, blood urea nitrogen (UREA), CREA, URIC and glucose, lower HDL levels and a prevalence of smoking when compared with the control groups (all *P*<0.05).

**Table 1:** General characteristics of the ACS patient and control groups

Characteristics	Uygur (*n* = 932)			Han (*n* = 1234)		
	Control (*n* = 461)	ACS (*n* = 471)	*P*-value	Control (*n* = 687)	ACS (*n* = 547)	*P*-value
Age (years)	54.0±9.2	54.0±9.6	0.966	57.8±11.1	56.8±11.9	0.115
Male, n (%)	273 (59.2)	264 (56.1)	0.328	391 (56.9)	332 (60.7)	0.180
Height (cm)	166.2±8.0	167.7±7.7	0.064	168.0±7.5	168.2±7.6	0.748
Weight (kg)	76.2±12.9	76.6±12.9	0.578	70.3±11.8	70.2±12.7	0.511
BMI (kg/m^2^)	27.2±3.9	27.3±4.2	0.729	24.9±3.2	24.6±3.3	0.624
SBP (mmHg)	127±18	127±17	0.919	124±19	123±19	0.206
DBP (mmHg)	77±12	76±11	0.460	76±12	76±13	0.366
Smoking, n (%)	168 (36.4)	195 (41.4)	0.121	365 (53.1)	328 (60.0)	0.016
Drinking, n (%)	113 (24.5)	130 (27.6)	0.283	346 (50.4)	305 (55.8)	0.059
HTN, n (%)	215 (46.6)	209 (45.8)	0.807	315 (45.9)	244 (44.6)	0.663
DM, n (%)	104 (22.6)	70 (15.4)	0.005	120 (17.5)	103 (18.8)	0.537
WBC (*10^9^/L)	6.7±2.1	9.6±3.4	<0.001	6.6±2.1	9.3±3.6	<0.001
TG (mmol/L)	1.8±1.3	2.2±2.2	0.002	1.7±1.1	2.0±1.7	<0.001
TC (mmol/L)	4.3±1.0	4.6±1.3	<0.001	4.1±1.0	4.3±1.3	0.007
HDL (mmol/L)	1.1±0.3	1.0±0.3	<0.001	1.1±0.3	1.0±0.4	<0.001
LDL (mmol/L)	2.7±0.8	2.9±1.0	0.006	2.6±0.8	2.7±1.0	0.067
UREA (mmol/L)	5.4±1.7	5.6±2.0	0.130	5.4±2.4	5.8±2.5	0.007
CREA (μmol/L)	67.8±17.7	74.3±21.8	<0.001	68.7±17.2	76.0±22.4	<0.001
URIC (μmol/L)	297.0±84.3	321.0±84.9	<0.001	300.6±85.1	320.2±94.6	<0.001
Glucose (mmol/L)	5.7±2.3	8.3±3.6	<0.001	5.5±1.9	8.1±3.9	<0.001

The continuous variables are defined as the mean ± SD. Categorical variables are expressed as percentages

The *P* value of the continuous variables was calculated by the independent samples t-test. The *P* value of the categorical variables was calculated by the Chi-square test

*HTN* hypertension, *DM* diabetes mellitus, *BMI* body mass index, *SBP* systolic blood pressure, *DBP* diastolic blood pressure, *WBC* leukocyte, *TG* triglyceride, *TC* total cholesterol, *HDL* high-density lipoprotein-cholesterol, *LDL* low-density lipoprotein-cholesterol, *UREA* urea nitrogen, *CREA* Creatinine, *URIC* uric acid

### Frequencies of *CCN1* gene alleles and genotypes in ACS patients and controls

The distributions of the genotypes and allele frequencies for three SNPs (rs6576776, rs954353 and rs3753794) of the *CCN1* gene in the Uygur and Han populations are presented in Table 2. All analyzed SNPs were in the HWE (all *P*>0.05). Among the Uygur population, the frequencies of the rs6576776 C allele and the CC genotype were more common in ACS patients than they were in the controls (C allele, 25.3% vs. 18.3%, *P*<0.001, CC genotype, 6.4% vs. 3.0%, *P*=0.001). A further analysis of the rs6576776 dominant model (CC+CG vs. GG, 44.2% vs. 55.8%, *P*=0.001) and recessive model (CC vs. CG+GG, 6.4% vs. 93.6%, *P*=0.016) revealed significant differences between the two groups. While we did observe comparable distributions of the rs954353 and rs3753794 genotypes and alleles between the ACS patients and controls (all *P*>0.05), in the Han population, neither the distributions of the three SNPs genotype and allele nor the distributions of models exhibited a significant difference between the ACS patients and the control subjects (all *P*>0.05).

**Table 2:** Genotype and allele distributions in ACS patients and control subjects

Polymorphisms			Uygur					Han				
			Control (*n* = 461)	ACS (*n* = 471)	OR	95%CI	*P*-value	Control (*n* = 687)	ACS (*n* = 547)	OR	95%CI	*P*-value
rs6576776	Genotype	CC	14 (3.0)	30 (6.4)	2.493	1.295-4.802	0.001	40 (5.8)	26 (4.8)	0.797	0.477-1.333	0.68
		CG	141 (30.6)	178 (37.8)	0.589	0.301-1.153		230 (33.5)	181 (33.1)	1.211	0.712-2.058	
		GG	306 (66.4)	263 (55.8)	0.401	0.208-0.772		417 (60.7)	340 (62.2)	1.254	0.750-2.097	
	Dominant model	CC+CG	155(33.6)	208(44.2)	1.561	1.197-2.036	0.001	270 (39.3)	207 (37.8)	0.94	0.746-1.184	0.601
		GG	306(66.4)	263(55.8)				417 (60.7)	340 (62.2)			
	Recessive model	CC	14(3.0)	30(6.4)	0.46	0.241-0.880	0.016	40 (5.8)	26(4.8)	1.239	0.746-2.057	0.407
		CG+GG	447(97.0)	441(93.6)				647 (94.2)	521 (95.2)			
	Allele	C	169(18.3)	238(25.3)	0.664	0.532-0.829	<0.001	310 (22.6)	233 (21.3)	1.077	0.888-1.305	0.451
		G	753(81.7)	704(74.7)				1064 (77.4)	861 (78.7)			
rs954353	Genotype	AA	205 (44.5)	208 (44.2)	1.118	0.726-1.723	0.777	285 (41.5)	220 (40.2)	1.049	0.727-1.516	0.714
		GA	202 (43.8)	214 (45.4)	1.044	0.795-1.371		315 (45.9)	263 (48.1)	1.082	0.851-1.375	
		GG	54 (11.7)	49 (10.4)	0.894	0.581-1.378		87 (12.7)	64 (11.7)	0.953	0.660-1.376	
	Dominant model	GA+GG	256 (55.5)	263(55.8)	1.013	0.782-1.311	0.925	402 (58.5)	327 (59.8)	1.054	0.838-1.324	0.653
		AA	205 (44.5)	208 (44.2)				285 (41.5)	220 (40.2)			
	Recessive model	GG	54(11.7)	49 (10.4)	1.143	0.758-1.722	0.524	87 (12.7)	64 (11.7)	1.094	0.776-1.544	0.608
		AA+GA	407 (88.3)	422 (89.6)				600 (87.3)	483 (88.3)			
	Allele	A	612 (66.4)	630 (66.9)	0.978	0.806-1.185	0.818	885 (64.4)	703 (64.3)	1.007	0.853-1.188	0.938
		G	310(33.6)	312 (33.1)				489 (35.6)	391 (35.7)			
rs3753794	Genotype	GG	187 (40.6)	190 (40.3)	1.227	0.809-1.860	0.429	298 (43.4)	238 (43.5)	0.849	0.579-1.243	0.556
		GA	210 (45.6)	228 (48.4)	1.311	0.871-1.974		321 (46.7)	245 (44.8)	0.811	0.555-1.185	
		AA	64 (13.9)	53 (11.3)	0.8115	0.538-1.236		68 (9.9)	64 (11.7)	1.178	0.805-1.726	
	Dominant model	AA+GA	274 (59.4)	281 (59.7)	1.009	0.777-1.311	0.944	389 (56.6)	309 (56.5)	0.995	0.793-1.248	0.963
		GG	187 (40.6)	190 (40.3)				298 (43.4)	238 (43.5)			
	Recessive model	AA	64 (13.9)	53 (11.3)	0.787	0.533-1.161	0.226	68 (9.9)	64 (11.7)	1.206	0.840-1.731	0.309
		GA+GG	397 (86.1)	418 (88.7)				619 (90.1)	483 (88.3)			
	Allele	A	338 (36.7)	334 (35.7)	1.043	0.863-1.261	0.662	457 (33.3)	373 (34.1)	0.963	0.814-1.140	0.663
		G	584 (63.3)	602 (64.3)				917 (66.7)	721 (65.9)			

*P* values for genotype and allele frequency distribution

*OR* odds ratio, *CI* confidence interval

### Association among *CCN1* gene linkage disequilibrium and haplotype analysis and susceptibility to ACS in Uygur population

**Fig. 1:**
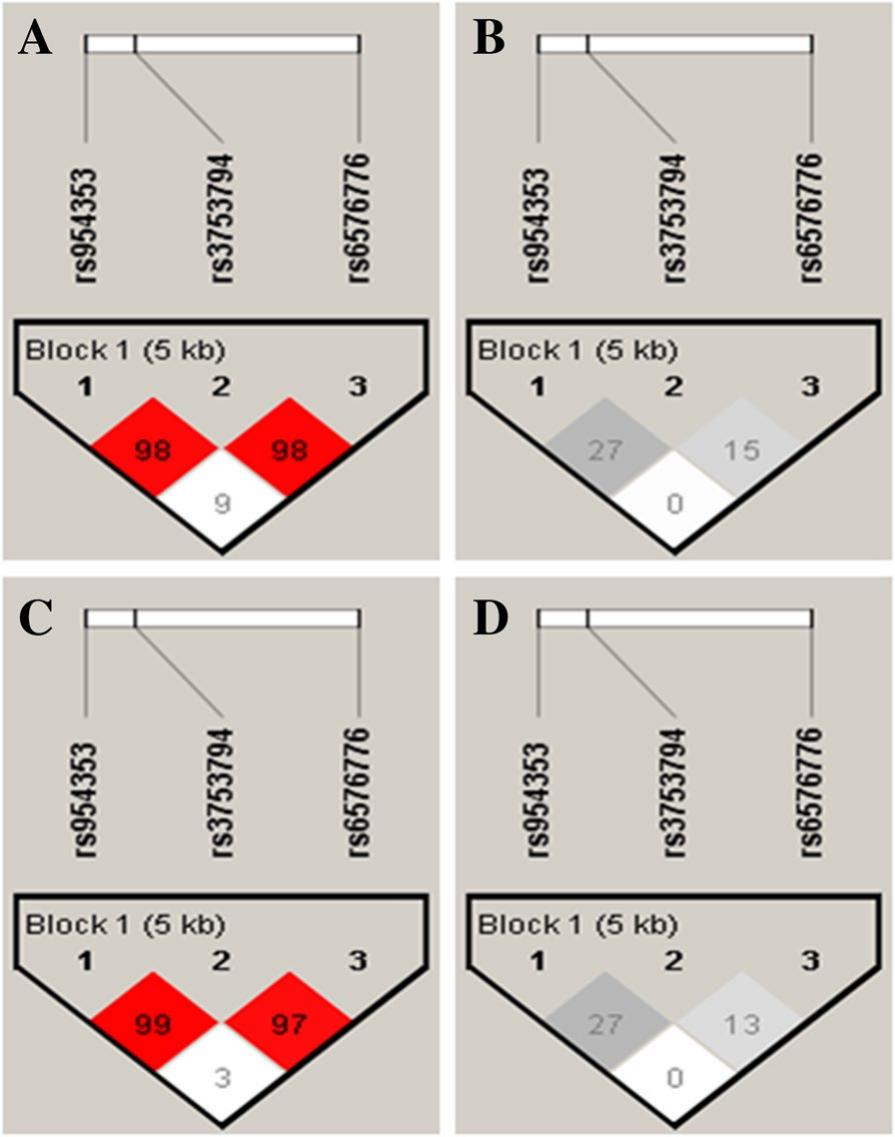
Pairwise linkage disequilibrium (|D'| above diagonal and r^2^ below diagonal) for the three polymorphisms. **a** |D'| and different colors represent different degree of linkage disequilibrium In Han population. A darker color corresponded to a stronger degree of linkage disequilibrium. **b** r^2^ In Han population. **c** |D'| and different colors represent different degree of linkage disequilibrium In Uygur population. **d** r^2^ In Uygur population

A haplotype analysis and a linkage disequilibrium indicated that rs6576776, rs954353 and rs3753794 were located in one haplotype block in both the Uygur and Han populations (Fig. 1). We also constructed eight haplotypes from the three SNPs of the *CCN1* gene using the Haploview 4.2 software. Among them, we excluded the GAC, AAC and GAG haplotypes because their frequencies were less than 0.01. Thus, we ultimately had five haplotypes in the Uygur and Han populations (Table 3). In the Uygur population, the frequencies of the GGC and AGC haplotypes were significantly higher in the ACS patients than they were in the control subjects (*P*=0.03, *P*=0.01, respectively). However, there were no significant differences in haplotype frequencies between the ACS patients and the control subjects in the Han population.

**Table 3:** Haplotype analyses in ACS patients and control subjects

LOCUS	Uygur						Han			
	Haplotype		Control	ACS	*P*-value	OR (95%CI)	Control	ACS	*P*-value	OR (95%CI)
SNP1| SNP2| SNP3	GGC	71.2 (7.7)		99.8 (10.6)	0.03	1.42 (1.035-1.958)	109.5 (8.0)	86.7 (7.9)	0.97	1.00 (0.747-1.344)
SNP1| SNP2| SNP3	AGC	97.2 (10.5)		138.8 (14.7)	0.01	1.47 (1.116-1.942)	199.2 (14.5)	144.4 (13.2)	0.35	0.90 (0.711-1.128)
SNP1| SNP2| SNP3	AAG	336.7 (36.5)		332.7 (35.3)	0.59	0.96 (0.780-1.164)	455.9 (33.2)	371.4 (34.0)	0.69	1.03 (0.874-1.224)
SNP1| SNP2| SNP3	GGG	238.5 (25.9)		210.9 (22.4)	0.08	0.83 (0.670-1.025)	379.1 (27.6)	305.2 (27.9)	0.86	1.01 (0.850-1.212)
SNP1| SNP2| SNP3	AGG	178.4 (19.4)		159.9 (17.0)	0.18	0.85 (0.675-1.081)	230.3 (16.8)	186.4 (17.0)	0.85	1.02 (0.824-1.260)

SNP1, rs954353; SNP2, rs3753794; SNP3, rs6576776

### *CCN1* gene rs6576776 C allele was a risk factor for ACS

A multiple logistic regression analysis concluded that the rs6576776 C allele (CC or CG genotype) was a factor in ACS susceptibility (OR=1.798, 95% CI=1.218-2.656, *P*=0.003) in the Uygur population after adjusting for age, smoking, drinking, lipids and liver function indicators, WBC, hypertension and DM (Table 4). In the Han population, we found that while the OR value of the rs6576776 C allele was 1.072, we did not observe a significant statistical difference with respect to the risk for ACS.

**Table 4:** Multiple logistic regression analysis for ACS patients and control subjects

Risk factor	Uygur			Han		
	OR	95%CI	*P*-value	OR	95%CI	*P*-value
rs6576776 CC+CG/GG	1.798	1.218-2.656	0.003	1.072	0.788-1.458	0.658
Smoking	1.214	0.736-2.001	0.447	0.859	0.536-1.375	0.526
Drinking	0.943	0.561-1.585	0.825	1.117	0.762-1.637	0.571
Hypertension	1.387	0.934-2.058	0.105	0.93	0.684-1.265	0.646
DM	0.587	0.348-0.990	0.046	1.474	1.008-2.157	0.046
WBC	1.261	1.122-1.417	<0.001	1.301	1.195-1.417	<0.001
CREA	1.012	1.000-1.024	0.045	1.013	1.003-1.023	0.011
URIC	0.999	0.996-1.001	0.357	0.998	0.996-1.000	0.034
TG	1.083	0.907-1.292	0.380	0.951	0.834-1.084	0.450
TC	1.25	0.813-1.922	0.308	1.548	1.181-2.028	0.002
HDL	0.33	0.144-0.759	0.009	0.078	0.042-0.145	<0.001
LDL	0.875	0.532-1.437	0.597	0.744	0.539-1.026	0.071

*OR* odds ratio, *CI* confidence interval, *DM* diabetes mellitusm, *WBC* leukocyte, *CREA* creatinine, *URIC* uric acidm, *TG* triglyceridem, *TC* total cholesterol, *HDL* high-density lipoprotein-cholesterol, *LDL* low-density lipoprotein-cholesterol

### Study power

Our data demonstrated that the probability of exposure to the *CCN1* gene rs6576776 CC and CG genotype in the Han and Uygur control groups was 0.393 and 0.336, respectively, while the OR of the ACS patients relative to control subjects among the Han and Uygur participants was 1.072 and 1.798, respectively. The power and sample size program revealed that the powers of our study were 0.092 in the Han population and 0.993 in the Uygur population.

## Discussion

At present, the global burden of cardiovascular disease has shifted toward low- and middle-income countries, including China, wherein over 80% of the global cardiovascular deaths occur. The etiology and pathogenesis of ACS are likely representative of a multifactorial disorder resulting from the inheritance of several susceptibility genes as well as from multiple environmental determinants [18]. We noted that the different ethnicities in Xinjiang may be a confounding factor of the present study and that the genetic backgrounds of these different ethnicities may help explain the mechanism behind ACS. To our knowledge, no study has addressed the association between *CCN1* SNPs and ACS. In this study, three SNPs of the *CCN1* gene, namely, rs6576776, rs954353 and rs3753794, were successfully genotyped in all ACS patients and controls of the Uygur and Han populations. We have demonstrated that (i) the frequencies of the CC genotypes and the C allele of rs6576776 were higher among ACS patients in the Uygur population; (ii) the three SNPs were located in one haplotype and in LD, and the frequencies of the GGC and the AGC haplotypes were significantly higher in ACS patients than in the control subjects, which led to a higher susceptibility to ACS in the Uygur population; and (iii) the rs6576776 C allele increased the risk of ACS in the Uygur subjects.

*CCN1*, which belongs to the CCN family is essential for cardiovascular development [19–21]. To date, there are no reports indicating a relationship between *CCN1* gene SNPs and susceptibility to ACS. However, it has been reported that *CCN1* gene expression is significantly increased in ApoE^-/-^ rat aorta and human carotid artery atherosclerotic plaques and that the *CCN1* gene may be involved in monocyte adhesion and migration during atherosclerosis, wherein activated monocytes adhere to CCN1 in a dependent manner through integrin αMβ2 and thereby accelerate monocyte migration to the vascular wall and promote the formation of atherosclerotic plaques [22, 23]. Andreas et al. sequenced *CCN1* exons in 143 patients with atrial septal defect (ASD) and found an extremely rare heterozygous missense mutation (c.139 C>T, p. R47W) in a severe ASD patient, which led to a completely different residue exchange at the highly conserved site of the N-terminal insulin-like growth factor binding protein module. Further validation studies confirmed that this variation is related to ASD [16]. Fan et al. showed that the *CCN1* gene deletion may impair cardiac valvuloseptal morphogenesis in mice, which leads to severe atrioventricular septal defects, because the deletion of *CCN1* can lead to premature apoptosis of cells at the atrial junction of endocardial cushion tissue and to reduce gelatinase activity in the interventricular septum muscle components [21].

It was reported that five *CCN1* SNPs (rs3753794, rs3753793, rs2297140, rs2297141 and IVS2150) were associated with plasma HDL cholesterol levels in obese individuals and that the risk of low HDL-C levels in patients carrying the A allele for rs3753794 increased by 1.56 fold [13]. The incidence of CCN1 rs6576776 C allele was about 25.3% in Chinese Uygur in our study, and NCBI database recorded 1000 Genomics were: 9.8% in Africa, 26.4% in East Asian, 13.8% in South Asian, 26.2% in Europe, and 23.3% in American (https://www.ncbi.nlm.nih.gov/snp/rs6576776#frequency_tab). Furthermore, the *CCN1* gene rs3753793 TG and the GG genotype were significantly correlated with prostate cancer, the distribution of the GG and TG genotypes was 3.85% and 50.00% [17]. Given the importance of *CCN1* in the biology of a variety of diseases, we investigated the relation of between the *CCN1* gene and ACS and found that Uygur individuals carrying the CC or CG genotypes of rs6576776 increased the incidence of ACS, thus indicating that the C allele is the risk factor for ACS. Our study also found that the frequencies of distribution of the GGC and AGC haplotypes among the ACS group of Uygur subjects were significantly higher than those in the control group, which may also be a potential cause of ACS, suggesting that the *CCN1* gene variation is a functional mutation. Therefore, together with the afore mentioned studies, these findings clearly indicate the *CCN1* gene polymorphism is more likely to be an independent risk factor for ACS susceptibility among the Uygur population.

There are some limitations in our study. First, as it was a single-center study, the results are less convincing than those of clinical trial studies. Hence, a multicenter study would be required to confirm this hypothesis. Second, only 1018 ACS patients were enrolled in this work, among which only 471 individuals from the Uygur population had ACS, a limitation that may affect the reliability of the results. Finally, as strategies were required to enhance the enrollment of the Uygur population and other ethnicities in the case-control studies, we cannot exactly pinpoint the potential mechanism and functional significance of the *CCN1* gene polymorphism on the risk of ACS.

## Conclusions

Our study documented that the *CCN1* gene rs6576776 C allele is associated with a higher risk of ACS and that the frequencies of GGC and AGC haplotypes constructed with rs6576776, rs954353 and rs3753794 are higher among the Uygur ACS patients.

## Methods

### Ethics

This study was approved by the Ethics Committee of the First Affiliated Hospital of Xinjiang Medical University, and, was based on the standards of the Helsinki Declaration. All participants provided informed consent, permission for DNA analyses, and consent for the collection of relevant clinical data.

### Study population

This case-control study consisted of 1234 Han (547 ACS patients and 687 controls) and 932 Uygur (471 ACS patients and 461 controls) who had visited the First Affiliated Hospital of Xinjiang Medical University between 2015 and 2018. The age and gender of the control group and the ACS group were matched. The inclusion criteria of ACS included: 1) The patients presented with persistent chest pain for more than 20 min, typical electrocardiographic changes including new pathologic Q waves and ST segment elevation of more than 1 mm, and increased plasma levels of creatinine kinase-MB isoenzyme (CK-MB) (more than two-fold higher than the upper reference limit) and/or troponin-T more than 0.1 μg/ml, according to the ACCF/AHA guidelines [24, 25]; 2) All ACS participating patients underwent coronary angiography to confirm the ACS diagnosis, exemplified with identifiable culprit-vessel, i.e., ≥50% luminal stenosis in at least one coronary artery or major branch segment. The inclusion criteria of control group included: Subjects who had no history of cardiovascular diseases and had no evidence of other systemic diseases as determined by history, blood test, ECG, and physical examination. The exclusion criteria included: Patients with echocardiographic-related valve abnormalities, local wall motion abnormalities, acute inflammatory diseases, autoimmune disease, severe liver or kidney dysfunction, or tumors. We chose appropriate therapy methods according to guideline [24, 25], patients were generally treated with standard medical therapy including lipid control, antiplatelet, anticoagulation, resistance to ischemia therapy and operative treatment including percutaneous coronary intervention and coronary artery bypass graft, etc.

### Definition of risk factors

Body mass index (BMI) was calculated by dividing the body weight (in kilograms) by the height in meters squared [26]. Individuals who admitted regular tobacco consumption in the past six months were considered current smokers [27]. Hypertension was defined as systolic blood pressure (SBP) exceeding 140 mmHg and/or diastolic blood pressure (DBP) greater than 90 mmHg on at least two different occasions [28]. Diabetes (DM) was defined as a history of diabetes, a random glucose value >11.1 mmol/L (200 mg/dl) on one occasion, and/or a fasting plasma glucose level >7.0 mmol/L (126 mg/dl) on two separate occasions or a 2-h plasma glucose ≥11.1 mmol/L during an oral glucose tolerance test plus signs or symptoms of DM [29].

### Biochemical assays

Standard venipuncture techniques and tubes containing ethylene diamine tetra-acetic acid were used in blood samples collected from all participants. Routine biochemical indicators were assessed by obtaining fasting peripheral blood samples (five mL), which were centrifuged at 3000 rpm for ten min to separate the plasma from the blood cells at 4°C. We measured the plasma concentrations of WBC, TG, TC, LDL, CREA, URIC, and glucose levels, a lower HDL level and DM total cholesterol (TC), triglycerides (TG), low level of LDL-C, high level of HDL-C, UREA, CREA, URIC, and glucose using standard methods as determined by a fully automatic biochemical analyzer (Beckman Coulter AU5800, US), while the leukocyte (WBC) was analyzed using the Coulter LH 750 analyzer (Beckman Coulter, Inc.) in the clinical laboratory of the First Affiliated Hospital, Xinjiang Medical University.

### Polymorphism selection

A minor allele frequency (MAF) ≥0.05 and linkage disequilibrium patterns with r^2^ >0.8 were used as cut offs by the Haploview software (Version 4.2) to select tagged SNPs according to the HapMap human SNP database (www.hapmap.org). Finally, we selected three SNPs located in different areas of the human *CCN1* gene (rs954353 in the intron, rs3753794 in the upstream transcript and rs6576776 in the downstream transcript).

### Genotyping of the *CCN1* gene

The standard phenol-chloroform method was used to extract DNA from leukocytes in the peripheral blood, and the DNA was stored at -80°C for further analysis. The SNPscan^TM^ genotyping assay (Genesky Biotechnologies Inc., Shanghai, China) was used to genotype the polymorphisms of the *CCN1* gene. Three probes were designed for each polymorphism: a two strip five-terminal identification probe and a one strip three-terminal probe. The five-terminal probe sequence consists of a universal primer sequence and an allele identification linkage sequence. The three-terminal probe sequence is the identification linkage sequence of polymorphism. The primers were synthesized by Genesky Biotechnologies Inc., Shanghai, China and their detailed sequences are presented in Table 5.

**Table 5:** *CCN1* genotyping primer sequence

Polymorphism	Primer name	Primer sequence
rs954353	Identification primer	5’-GTGTACGTGTTGGGCCCTCA-3’
	Identification primer	5’-GTGTACGTGTTGGGCCCTCG-3’
	Universal primer	5’-CTTTCCTCACTGTATTTGCATAACACC-3’
rs3753794	Identification primer	5’-GGGGGAGACCTCTGCCTTGG-3’
	Identification primer	5’-GGGGGAGACCTCTGCCTCGA-3’
	Universal primer	5’-AATTTGCCAGACGATGGGCA-3’
rs6576776	Identification primer	5’-TGGCCCTCACGCTATTGGAAAAGAAG-3’
	Identification primer	5’-TGGCCCTCACGCTATTGGAAAAGAAC-3’
	Universal primer	5’-TTGAAATTCCTTGCATTCCTTTGC-3’

### Statistical analysis

Data analyses were performed using the SPSS22.0 software package (SPSS, Inc., Chicago, IL, USA). Continuous variables were expressed as the mean ± standard deviation (SD). An independent sample t-test was used to compare differences between the ACS groups and the control groups. Categorical variables were presented as a number or proportions. Differences between genotypic and allelic frequencies as well as Hardy-Weinberg equilibrium (HWE) were analyzed using the χ^2^ test. Haploview 4.2 was used to reconstruct haplotypes, and the plink program was used to obtain haplotypes and haplotype frequencies and was further used for haplotype association studies [30]. The association between *CCN1* gene polymorphisms and ACS was analyzed via multiple logistic regression. The power of this study was calculated using the Power and Sample Size Program (Version 3.0.43), and statistical significance was set at *P*<0.05 (two-tailed).

## Data Availability

The datasets analyzed during the current study including accession information for the raw genotyping data are available from the corresponding author upon reasonable request.

## Abbreviations

CAD: Coronary artery disease
ACS: Acute coronary syndrome
CCN1: Cysteine-rich 61
ECM: Extracellular matrix
WBC: Leukocyte
TG: Triglycerides
TC: Total cholesterol
LDL-C: Low density lipoprotein-cholesterol
CREA: Creatinine
URI-C: Uric acid
HDL-C: High density lipoprotein-cholesterol
UREA: Blood urea nitrogen
CK-MB: Creatinine kinase-MB isoenzyme
BMI: Body mass index
SBP: Systolic blood pressure
DBP: Diastolic blood pressure
DM: Diabetes
MAF: Minor allele frequency

## References

[1] McAloon CJ, Boylan LM, Hamborg T, Stallard N, Osman F, Lim PB (2016). The changing face of cardiovascular disease 2000-2012: an analysis of the world health organisation global health estimates data. Int J Cardiol.

[2] Raaz D, Herrmann M, Ekici AB, Klinghammer L, Lausen B, Voll RE (2009). FcgammaRIIa genotype is associated with acute coronary syndromes as first manifestation of coronary artery disease. Atherosclerosis.

[3] Wu P, Ma G, Li N (2017). The profile of Cyr61 expression data correlate to the skin inflammation in psoriasis. Data Brief.

[4] Zhao JF, Chen HY, Wei J, Jim Leu SJ, Lee TS (2019). CCN family member 1 deregulates cholesterol metabolism and aggravates atherosclerosis. Acta Physiol (Oxf).

[5] Bleau AM, Planque N, Perbal B (2005). CCN proteins and cancer: two to tango. Front Biosci.

[6] Papetta A, Gakiopoulou H, Agapitos E, Patsouris ES, Lazaris AC (2013). Correlations between CCN1 immunoexpression and myocardial histologic lesions in sudden cardiac death. Am J Forensic Med Pathol.

[7] Deng J, Qian X, Li J, Li Y, Li Y, Luo Y (2018). Evaluation of serum cysteine-rich protein 61 levels in patients with coronary artery disease. Biomark Med.

[8] Zhao J, Zhang C, Liu J, Zhang L, Cao Y, Wu D (2018). Prognostic significance of serum cysteine-rich protein 61 in patients with acute heart failure. Cell Physiol Biochem.

[9] Bonda TA, Kaminski KA, Dziemidowicz M, Litvinovich S, Kozuch M, Hirnle T (2012). Atrial expression of the CCN1 and CCN2 proteins in chronic heart failure. Folia Histochem Cytobiol.

[10] Bonda TA, Kozuch M, Litvinovich S, Bialuk I, Taranta A, Lipiec P (2015). Transcriptional and post-transcriptional regulation of CCN genes in failing heart. Pharmacol Rep.

[11] Bonda TA, Taranta A, Kaminski KA, Dziemidowicz M, Litvinovich S, Kozuch M (2013). CCN1 expression in interleukin-6 deficient mouse kidney in experimental model of heart failure. Folia Histochem Cytobiol.

[12] Jay P, Berge-Lefranc JL, Marsollier C, Mejean C, Taviaux S, Berta P (1997). The human growth factor-inducible immediate early gene, CYR61, maps to chromosome 1p. Oncogene.

[13] Bouchard L, Tchernof A, Deshaies Y, Lebel S, Hould FS, Marceau P (2007). CYR61 polymorphisms are associated with plasma HDL-cholesterol levels in obese individuals. Clin Genet.

[14] Niu CC, Wan YF, Yang C, Li T, Liao P (2018). Polymorphisms of the CYR61 gene in patients with acute myeloid leukemia in a Han Chinese population. Medicine (Baltimore).

[15] Planck T, Shahida B, Sjogren M, Groop L, Hallengren B, Lantz M (2014). Association of BTG2, CYR61, ZFP36, and SCD gene polymorphisms with Graves’ disease and ophthalmopathy. Thyroid.

[16] Perrot A, Schmitt KR, Roth EM, Stiller B, Posch MG, Browne EN (2015). CCN1 mutation is associated with atrial septal defect. Pediatr Cardiol.

[17] Tao L, Chen J, Zhou H, Qin C, Li P, Cao Q (2013). A functional polymorphism in the CYR61 (IGFBP10) gene is associated with prostate cancer risk. Prostate Cancer Prostatic Dis.

[18] Zheng YY, Xie X, Ma YT, Yang YN, Fu ZY, Li XM (2011). Relationship between a novel polymorphism of the C5L2 gene and coronary artery disease. PLoS One.

[19] Babic AM, Kireeva ML, Kolesnikova TV, Lau LF (1998). CYR61, a product of a growth factor-inducible immediate early gene, promotes angiogenesis and tumor growth. Proc Natl Acad Sci U S A.

[20] Mo FE, Muntean AG, Chen CC, Stolz DB, Watkins SC, Lau LF (2002). CYR61 (CCN1) is essential for placental development and vascular integrity. Mol Cell Biol.

[21] Mo FE, Lau LF (2006). The matricellular protein CCN1 is essential for cardiac development. Circ Res.

[22] Schober JM, Chen N, Grzeszkiewicz TM, Jovanovic I, Emeson EE, Ugarova TP (2002). Identification of integrin alpha(M)beta(2) as an adhesion receptor on peripheral blood monocytes for Cyr61 (CCN1) and connective tissue growth factor (CCN2): immediate-early gene products expressed in atherosclerotic lesions. Blood.

[23] Bai T, Chen CC, Lau LF (2010). Matricellular protein CCN1 activates a proinflammatory genetic program in murine macrophages. J Immunol.

[24] Jneid H, Anderson JL, Wright RS, Adams CD, Bridges CR, Casey DE (2012). 2012 ACCF/AHA focused update of the guideline for the management of patients with unstable angina/non-ST-elevation myocardial infarction (updating the 2007 guideline and replacing the 2011 focused update): a report of the American College of Cardiology Foundation/American Heart Association Task Force on Practice Guidelines. J Am Coll Cardiol.

[25] Levine GN, Bates ER, Blankenship JC, Bailey SR, Bittl JA, Cercek B (2016). 2015 ACC/AHA/SCAI Focused Update on Primary Percutaneous Coronary Intervention for Patients With ST-Elevation Myocardial Infarction: An Update of the 2011 ACCF/AHA/SCAI Guideline for Percutaneous Coronary Intervention and the 2013 ACCF/AHA Guideline for the Management of ST-Elevation Myocardial Infarction: A Report of the American College of Cardiology/American Heart Association Task Force on Clinical Practice Guidelines and the Society for Cardiovascular Angiography and Interventions. Circulation.

[26] Chen X, Gui G, Ji W, Xue Q, Wang C, Li H. The relationship between obesity subtypes based on BMI and cardio-cerebrovascular disease. Hypertens Res. 2019;42(6):912–9. 10.1038/s41440-018-0184-4 30622319

[27] Koene RJ, Prizment AE, Blaes A, Konety SH (2016). Shared risk factors in cardiovascular disease and cancer. Circulation.

[28] Mancia G (2014). The new American guidelines on hypertension: a favorable opinion. J Clin Hypertens (Greenwich).

[29] Luo JY, Xu R, Li XM, Zhou Y, Zhao Q, Liu F (2016). MIF gene polymorphism rs755622 is associated with coronary artery disease and severity of coronary lesions in a Chinese Kazakh population: a case-control study. Medicine (Baltimore).

[30] Purcell S, Neale B, Todd-Brown K, Thomas L, Ferreira MA, Bender D (2007). PLINK: a tool set for whole-genome association and population-based linkage analyses. Am J Hum Genet.

